# A Treatise on the Practice of Medicine

**Published:** 1887-02

**Authors:** 


A Treatise on The Practice of Medicine, for the use of Students and Practitioners of Medicine, By Robert Bartholow, M. A. M. D. L. L. D. Prof. Materia Medica, General Therapeutics and Hygiene in the Jefferson Medical College of Philadelphia and Dean of the faculty; formerly Professor of Theory and Practice of Medicine in the Medical College of Ohio, Etc.

Sixth edition, revised and enlarged, New York, 1886, D. Appelton & Co. 1, 3 and 5, Bond St., Cloth, $5.00.

This work has now been before the profession so long as to need no special attention at our hands. Its popularity is due in part to the prominence given to practical therapeutics and to symptomatology. It is too often that in the eager desire to know what to do for a patient, the doctor frequently does not enquire very thoroughly as to what is the matter with him; does not make a clear diagnosis based upon a thorough reading of the symptoms but goes to work. In the revision the matter introduced represents the advanced ideas and accepted facts of the times. It is a standard work and should be on every progressive Doctor's shelf.

